# Supplementary material for: Probing the active site in single-atom oxygen reduction catalysts via operando X-ray and electrochemical spectroscopy
Source: Nat Commun. 2020 Aug 25;11:4233. doi: 10.1038/s41467-020-17975-y (PMC7447817; doi:10.1038/s41467-020-17975-y)
Supplement: Supplementary file 1 — Supplementary Information [file 41467_2020_17975_MOESM1_ESM.pdf]

Supporting Information

Probing the Active Site in Single-atom Oxygen Reduction Catalysts via Operando X-ray and Electrochemical Spectroscopy

Hsiang-Ting Lien,[a,b,§] Sun-Tang Chang,[c,§] Po-Tuan Chen,[a,b,d§] Deniz P. Wong,[e] Yu-Chung Chang,[a,c,f] Ying-Rei Lu,[f,g] Chung-Li Dong,[g] Chen-Hao Wang,[c] Kuei-Hsien Chen,[a,e*] and Li-Chyong Chen[a,b*]

[§] H. T. Lien, S. T. Chang and P. T. Chen contributed equally to this work

[a] Center for Condensed Matter Sciences, National Taiwan University, Taipei, Taiwan
[b] Center of Atomic Initiative for New Materials, National Taiwan University, Taipei, Taiwan
[c] Department of Materials Science and Engineering, National Taiwan University of Science and Technology, Taipei, Taiwan
[d] Department of Vehicle Engineering, National Taipei University of Technology, Taipei, Taiwan
[e] Institute of Atomic and Molecular Sciences, Academia Sinica, Taipei, Taiwan
[f] National Synchrotron Radiation Research Center, Hsinchu, Taiwan
[g] Department of Physics, Tamkang University, Tamsui, Taiwan

*Corresponding authors:
Dr. Li-Chyong Chen: chenlc@ntu.edu.tw
Dr. Kuei-Hsien Chen: chenkh@pub.iams.sinica.edu.tw

Supplementary Figures

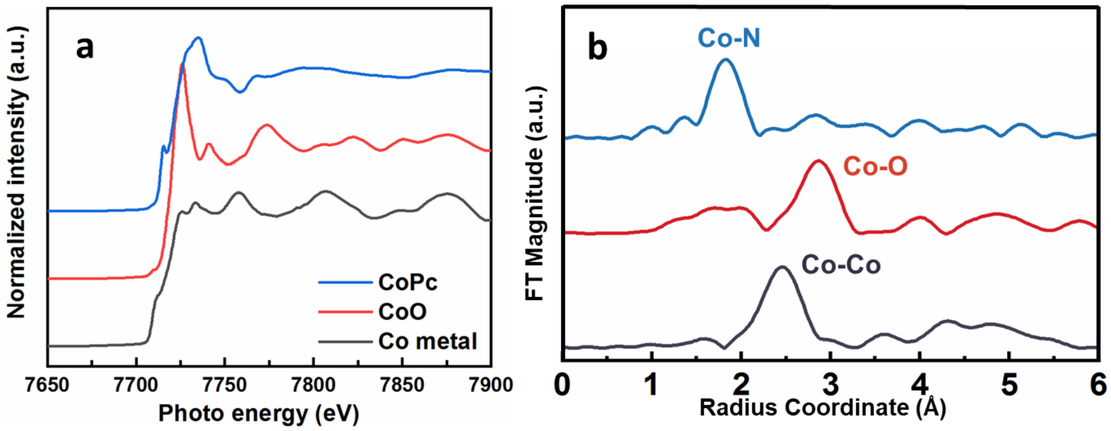

Supplementary Figure 1. Ex situ Co K edge XANES and EXAFS spectrum. (a) Co K edge XANES spectra of three different references, specifically CoPc, CoO and Co metal. (b) The corresponding phase-corrected Fourier transforms EXAFS (k3-weighted) from (a).

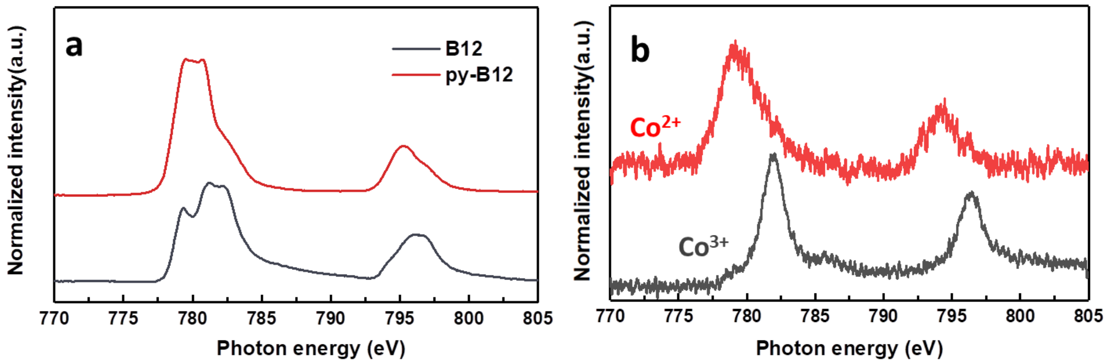

Supplementary Figure 2. Ex situ Co L edge NEXAFS spectrum. Soft X-ray absorption Co $L_{3/2}$ edge spectra of py-B12 and pristine B12, exhibiting Co^{2+} and Co^{3+}, respectively. The signals were collected from different mode. (a) Total electron yield (TEY). (b) Total fluorescence yield (TFY).

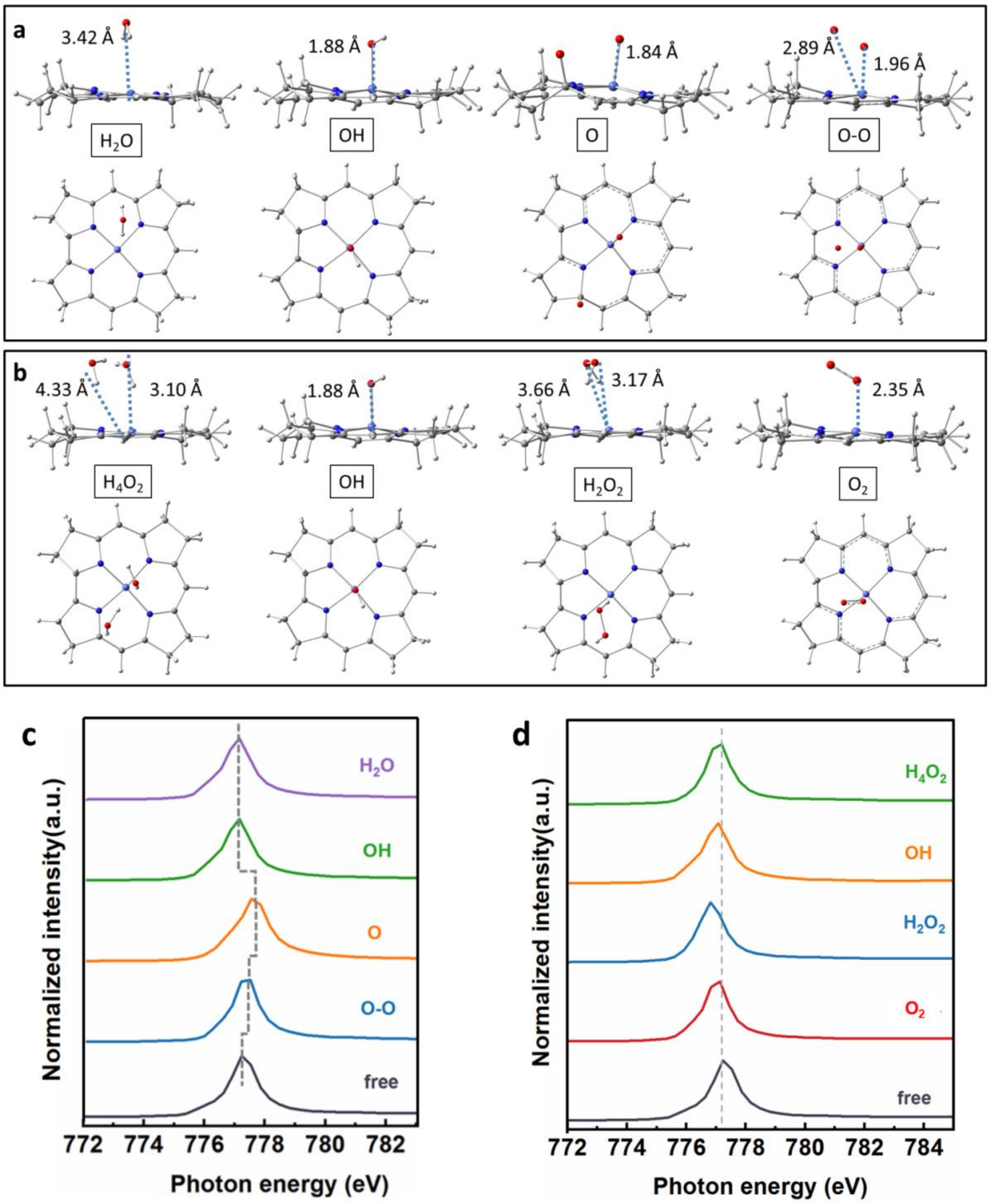

Supplementary Figure 3. Molecular Orbital Simulation and Co L_3 edge simulated spectrum. (a) Optimized structures for the models of four-electron process (OO, O, OH, and H_2O). (b) FEFF calculated Co L edge with the different oxygen species of four-electron process. (c) Optimized structures for the models of two-electron process (O_2, H_2O_2, OH, and H_4O_2). (b) FEFF calculated Co L edge with the different oxygen species of two-electron process. If the reaction went through two-electron process, there would be no redshift in the XANES spectrum. XANES calculations were performed using DFT-optimized structures to theoretically examine Co L-edge XANES spectra related to oxygen-based adsorbates on the Co–corrin cluster. XANES calculations of Co were performed by the FEFF8 code. The

self-consistent potential and full multiple scattering were calculated at a 5.0-Å radius. In the initial input, a negative charge is equally allocated on every atom of a cluster. After self-consistent calculation, the charge would be redistributed correctly. To compare the experimental and calculated spectra, a rigid shift of 2 eV to higher energy was applied to each calculated spectrum.

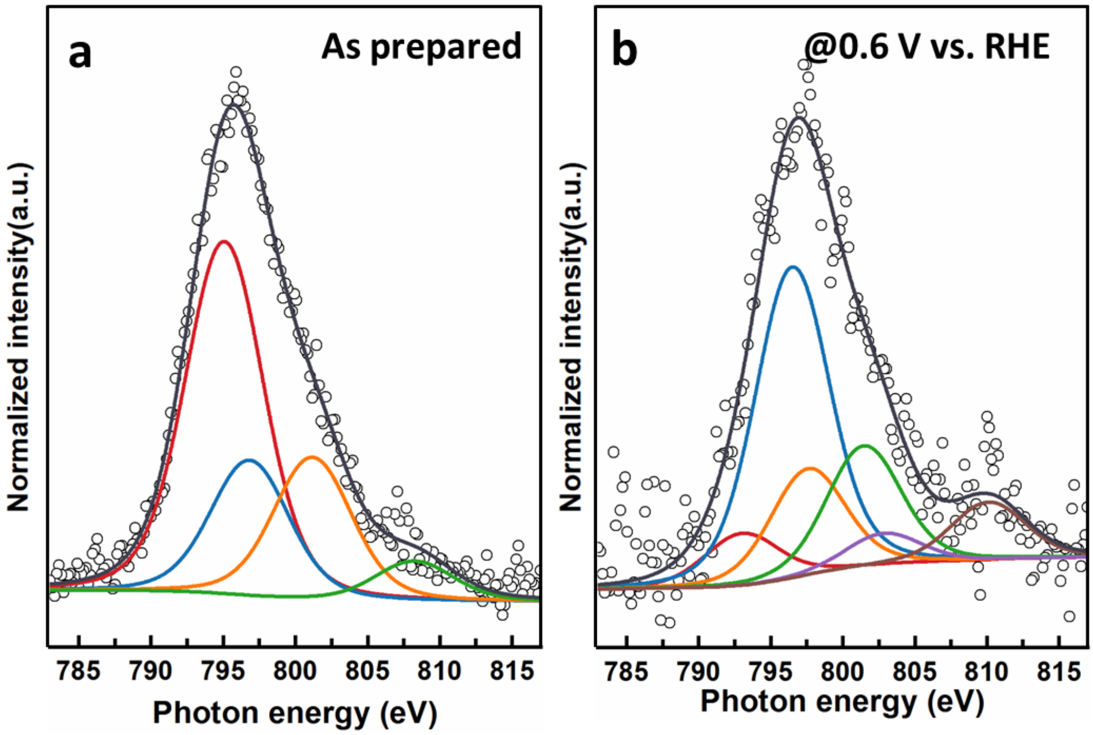

Supplementary Figure 4. The Co L_3 edge spectrum fitting. Soft X-ray absorption of Co L_3 edge spectra of the as prepared py-B12 and under operando potential (@ 0.6V), respectively. The peak fitting results were summarized in **Supplementary Table 2**.

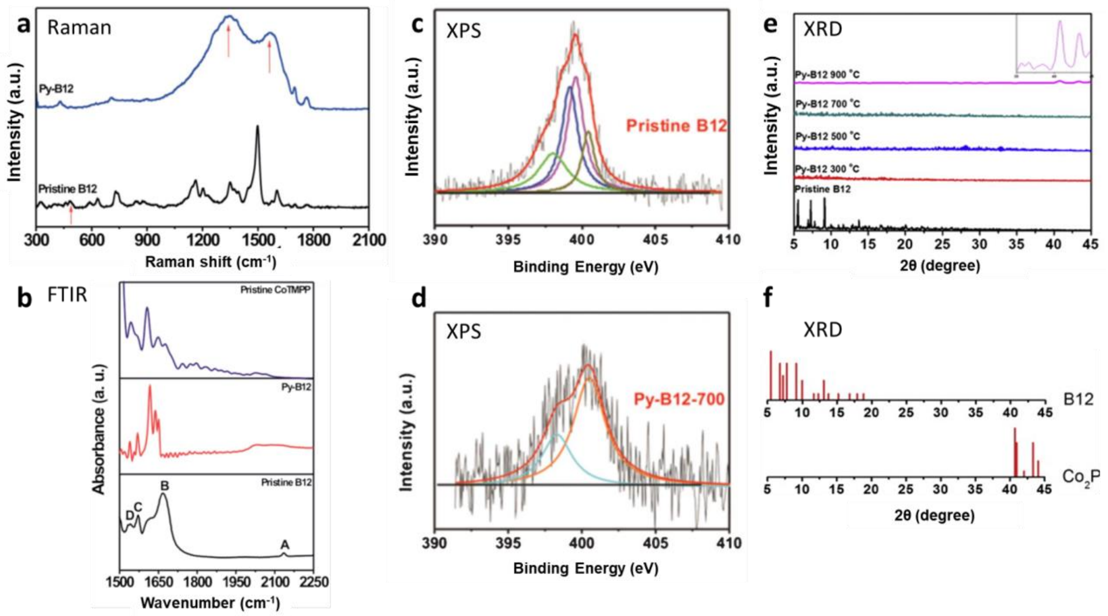

Supplementary Figure 5. Ex-situ characterizations of electrocatayst. (a) Raman of py-B12 and pristine B12 [1], (b) FTIR of py-B12 and pristine B12 [1], (c) XPS of pristine B12 [2], (d) XPS of py-B12 and pristine B12 [2], (e) XRD of py-B12 and pristine B12 [3], and (f) XRD of B12 and Co_2P [3]. In Raman spectra, the two strong peaks at 1330 and 1580 cm^{-1} are attributed to D- and G-peaks, respectively, of the carbon-like materials, suggesting that py-B12 forms a network structure of poly-aromatic hydrocarbons. Similar comparison of the FTIR spectra shows four characteristic peaks (A to D) for pristine B12. XPS suggested that the pyrolysis converts the nitrogen into pyridinic-like nitrogen and quaternary N-type nitrogen, with N^+ at 398.7 eV and 401.4 eV. XRD presented that pristine B12 exhibits the same characteristic peaks as observed in the vitamin B12 reference pattern.

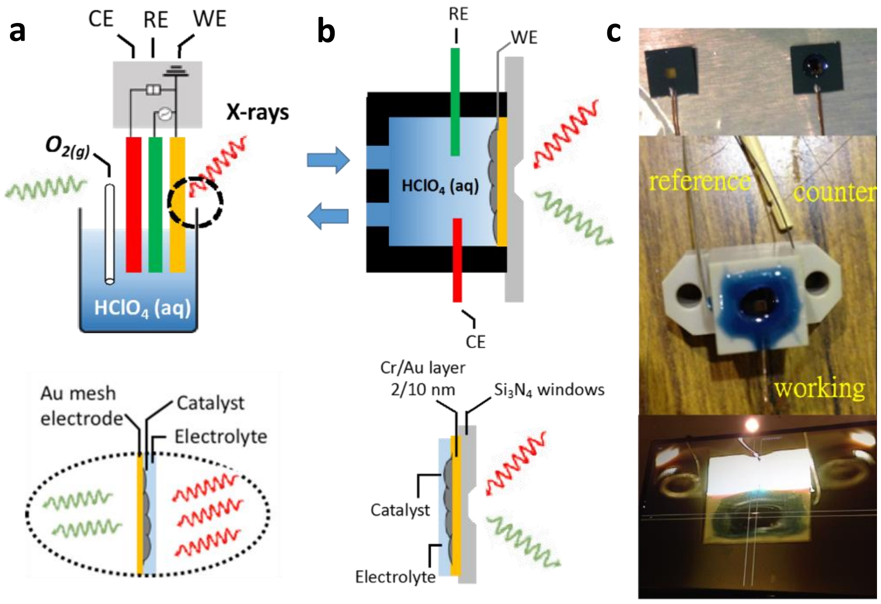

Supplementary Figure 6. Schematic diagram of in-situ spectroelectrochemical setups. (a) hard XAS in transmission mode and (b) flow cell for soft XAS in fluorescence mode. (c) Photo for the actual setup used in soft XAS.

Supplementary Tables

Supplementary Table 1. The equivalent circuits fitting results. Analyses of the electrochemical impedance spectra with the respective equivalent circuits proposed in different operation conditions. The fitting elements were assigned to double-layer capacitance of catalyst layer (C_{DL}), the charge transfer resistance (R_{ct}), mass-transfer resistance (R_{mt}) and capacitance of reaction process (C_{rxn}). Warburg impedance takes into account a finite diffusion. M is restricted linear ordinary diffusion impedance element with a reflective boundary.

Potential (V vs. RHE)	model	R_c (Ω)	C_{DL} (F)	R_{ct} (Ω)	R_{mt} (Ω)	C_{rxn} (F)	W(Ω)	M(Ω)
1.2 V vs. RHE	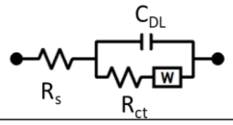	16.44	2.82E-3	225.4	--	--	38.05	--
1.0 V vs. RHE	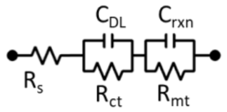	16.44	2.82E-3	222.4	153.7	0.03	--	--
0.8 V vs. RHE	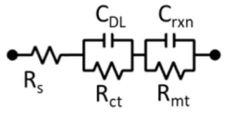	16.44	2.82E-3	124.8	52.05	0.683	--	--
0.6 V vs. RHE	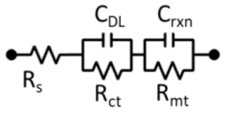	16.44	2.81E-3	68.22	21.6	0.524	--	--
0.4 V vs. RHE	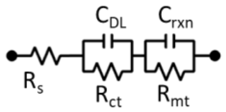	16.44	2.79E-3	44.14	2.65	0.514	--	--
0.2 V vs. RHE	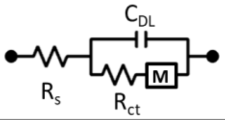	16.44	2.80E-4	30.95	--	--	--	33.39

Supplementary Table 2. The molecular orbital energy-level diagram. The d^7 electron D_{4h}/S_4 symmetry crystal field orbital and a hybridization orbital fitting between pre-catalytic and catalytic condition.

Fitting result	As prepared	@ 0.6 V vs. RHE
$\boldsymbol{d_{x^2-y^2}}$	808.22	810.09
$\boldsymbol{\sigma^*}\ (\boldsymbol{d_{xy}} + \boldsymbol{\sigma_{2p}})$	--	802.81
$\boldsymbol{\pi^*_{2p}}$	--	801.46
$\boldsymbol{\sigma}(\boldsymbol{d_{xy}} + \boldsymbol{\sigma_{2p}})$	--	797.57
$\boldsymbol{d_{xy}}$	801.18	--
$\boldsymbol{d_{z^2}}$	796.84	796.50
$\boldsymbol{d_{yz}}, \boldsymbol{d_{xz}}$	795.09	793.02

Supplementary References

[1] Chang, S.-T. et al. Vitalizing fuel cells with vitamins: pyrolyzed vitamin B12 as a non-precious catalyst for enhanced oxygen reduction reaction of polymer electrolyte fuel cells. *Energy Environ. Sci.* **5**, 5305–5314 (2012).

[2] Wang, C.-H. et al. High stability pyrolyzed vitamin B12 as a non-precious metal catalyst of oxygen reduction reaction in microbial fuel cells. *RSC Adv.* **3**, 15375–15381 (2013).

[3] Chang, S.-T. et al. Preparation of non-precious metal catalysts for PEMFC cathode from pyrolyzed vitamin B12. *Int. J. Hydrog. Energy* **37**, 13755–13762 (2012).
